# A critical role for NF2 and the Hippo pathway in branching morphogenesis

**DOI:** 10.1038/ncomms12309

**Published:** 2016-08-02

**Authors:** Antoine Reginensi, Leonie Enderle, Alex Gregorieff, Randy L. Johnson, Jeffrey L. Wrana, Helen McNeill

**Affiliations:** 1 Lunenfeld-Tanenbaum Research Institute, Mount Sinai Hospital, Toronto, Ontario, Canada M5G 1X5; 2 Department of Biochemistry & Molecular Biology, University of Texas MD Anderson Cancer Center, Houston, Texas 77030, USA; 3 Department of Molecular Genetics, University of Toronto, Toronto, Ontario, Canada M5G 1X5

## Abstract

Branching morphogenesis is a complex biological process common to the development of most epithelial organs. Here we demonstrate that NF2, LATS1/2 and YAP play a critical role in branching morphogenesis in the mouse kidney. Removal of *Nf2* or *Lats1*/*2* from the ureteric bud (UB) lineage causes loss of branching morphogenesis that is rescued by loss of one copy of *Yap* and *Taz*, and phenocopied by YAP overexpression. Mosaic analysis demonstrates that cells with high YAP expression have reduced contribution to UB tips, similar to *Ret*^*−/−*^ cells, and that YAP suppresses RET signalling and tip identity. Conversely, *Yap*/*Taz* UB-deletion leads to cyst-like branching and expansion of UB tip markers, suggesting a shift towards tip cell identity. Based on these data we propose that NF2 and the Hippo pathway locally repress YAP/TAZ activity in the UB to promote subsequent splitting of the tip to allow branching morphogenesis.

## Abstract (web summary)

Branching morphogenesis is essential for the formation of most epithelial organs. Here, the authors show that Neurofibromatosis 2 (NF2), the Hippo pathway kinases LATS1 and LATS2, and the transcriptional co-activators YAP and TAZ control tip identity, RET signalling and branching morphogenesis in the mouse kidney.

## Graphical abstract

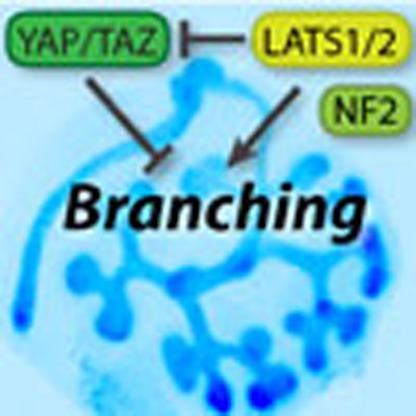

Branching morphogenesis is critical for the development and function of most epithelial organs, and is essential for the formation of the mammalian kidney. The kidney develops through reciprocal signalling between a ureteric epithelium that forms the collecting duct, and surrounding mesenchymal nephron progenitors and stroma^1^. The bilateral symmetry and characteristic shape of kidneys indicates branching morphogenesis is highly controlled. In addition, the rotational angle between one set of branches and the next is relatively fixed implying tight regulation^2^. How this developmentally critical branching is so tightly regulated is still unclear.

The first step of kidney development occurs when the ureteric bud (UB) invades the metanephric mesenchyme. Tip identity is defined in response to metanephric mesenchyme-derived signals including Glial cell-line-derived neurotrophic factor (GDNF) and fibroblast growth factors^3^. The tip contains progenitor cells that can self-renew or be left behind to give rise to trunk cells^4^. Binding of GDNF to its receptors GFRα1 and RET, triggers tyrosine kinase signalling, which induces the outgrowth of the UB. Once the ureter has invaded the metanephric mesenchyme, GDNF/RET signalling at UB tips leads to growth and repetitive branching of the ureter to form a ureteric tree that will give rise to the collecting duct. Loss of *Gdnf*, *Ret* or *Gfrα1* impairs branching morphogenesis causing kidney defects ranging from renal dysplasia to complete agenesis^5^,^6^,^7^. RET signalling is essential to form and maintain tips, and promotes a feed-forward signalling loop, in which RET signalling promotes expression of *Ret* transcript. The tip domain swells to form an ampulla before a symmetry breaking event occurs that allows it to split into two new tips. How symmetry is broken in the ampulla to form a branch is not understood.

*Neurofibromatosis 2* (*Nf2*) encodes a large FERM (4.1 protein/ezrin/radixin/moesin)-domain containing protein also known as MERLIN. Mutations in *NF2* are responsible for Neurofibromatosis type 2, a dominantly inherited tumour predisposition syndrome, characterized by the formation of benign neural tumours^8^,^9^,^10^. Despite extensive research, the mechanisms by which mutations in *NF2* cause disease remain unclear, due in part to multiple roles of NF2 in controlling several signalling pathways including PI3K-AKT, RAC-PAK, FAK-SRC and EGFR-RAS-ERK (ref. 10).

Studies both in flies and mammals suggest that NF2 can also regulate the Hippo pathway. The Hippo pathway is a conserved kinase cassette that regulates tissue growth by controlling the activity of YAP and TAZ (refs 11, 12, 13). YAP and TAZ are closely related transcriptional co-activators that promote the expression of pro-proliferative and anti-apoptotic genes. Upstream of YAP and TAZ are the Hippo kinases MST1/2 and LATS1/2, which negatively regulate YAP and TAZ and cause their exclusion from the nucleus. Loss of Hippo signalling leads to unrestricted proliferation in flies and mammals, and has been linked to a variety of developmental abnormalities and cancers^14^,^15^. NF2 can bind and recruit LATS to the plasma membrane, where it is activated by MST kinases^16^. NF2 has also been shown to bind other Hippo pathway components^8^. NF2 is one of many regulators of the Hippo pathway^17^: Cell adhesion, cell polarity, mechanical forces and the cytoskeleton have all been shown to regulate YAP localization in tissue culture^18^, suggesting that as tissues grow and develop, feedback may occur from resultant changes in the environment.

Here we uncover an unsuspected role for NF2 and the Hippo pathway in kidney branching morphogenesis. We find that *Nf2* conditional mutants have severe renal hypodysplasia due to defective branching morphogenesis. *Nf2* kidney hypodysplasia can be rescued by reducing *Yap* and *Taz* dosage, and phenocopied by YAP overexpression, suggesting that NF2 restricts YAP/TAZ activity to promote branching morphogenesis. *Lats1/2* deletion leads to kidney agenesis that can be rescued by reducing YAP/TAZ levels, suggesting that high YAP/TAZ activity inhibits branching. Importantly, loss of *Nf2*, or overexpression of YAP inhibits RET signalling and expression of tip markers. Our mosaic analysis reveals that YAP overexpressing cells show cell-autonomous loss of RET signalling and are excluded from UB tips. In contrast, we find that UB-specific deletion of *Yap* and *Taz* leads to cyst-like branching, associated with an expanded UB tip domain. Taken together, these data demonstrate an essential role for NF2 and the Hippo pathway in regulation of branching morphogenesis in the mammalian kidney.

## Results

### *Nf2* deletion results in severe kidney defects

**Figure 1:**
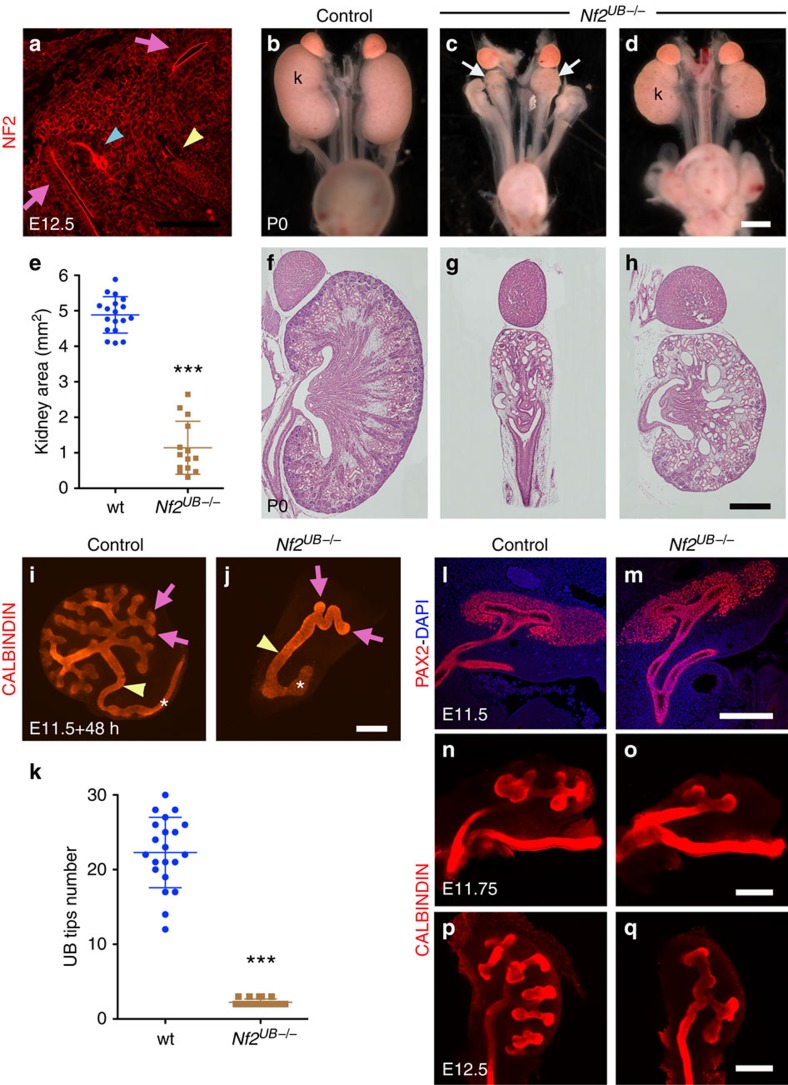
*Nf2* deletion leads to kidney defects due to defective branching morphogenesis. (**a**) NF2 protein is apically localized in all kidney epithelia: UB (pink arrows), collecting duct (yellow arrowhead) and early nephron (blue arrowhead). See Supplementary Fig. 1d for counterstaining with E-CADHERIN to mark the UB and early nephron. (**b**–**d**) Macroscopic view of the urogenital system from wild-type and *Nf2*^*UB−/−*^ mutants at P0 shows severe kidney hypoplasia (arrows) in *Nf2* mutants. (**e**) Quantification revealed a 76% decrease in kidney size in *Nf2* mutants (*n*=14 kidneys) compared with controls (*n*=9). Error bars represent s.d., ***P<0.0001, Student's *t*-test. (**f**–**h**) Periodic acid-Schiff (PAS) staining of P0 kidneys from wild type and *Nf2*^*UB−/−*^. (**i**,**j**) *Ex vivo* analysis demonstrates loss of ureter branching in *Nf2*^*UB−/−*^ compared with controls, visualized using anti-CALBINDIN antibody (pink arrows point to UB tips, yellow arrowheads to the ureter/collecting duct (CD) and asterisks mark the Wolffian duct). **(k**) Quantification of ureter branching capacity from *ex vivo* kidney explant experiments. For quantification, 21 kidneys explants were used in both genotypes. Error bars represent s.d., ***P<0.0001, Student's *t*-test. The *y* axis represents the number of ureteric tips after 48 h of culture. (**l**–**q**) Similar branching defects were observed *in vivo* using immunostaining on sections (PAX2 antibody, E11.5) and whole-mount immunostaining (CALBINDIN antibody, E11.75 and E12.5). Scale bars represent 100 μm (**a**), 1 mm (**b**–**d**), 0.5 mm (**f**–**h**), 250 μm (**i**,**j**) and 200 μm (**l**–**q**).

To investigate the role of NF2 during kidney development, we first stained developing kidneys with NF2 antibody, and found that NF2 protein uniformly localized along the apical membrane of the UB and nephrons throughout development (Fig. 1a and Supplementary Fig. 1a–h). NF2 is maintained in the adult kidney, although at a weaker level (Supplementary Fig. 1i). The apical staining is specific, as it is lost in *Nf2* mutant tissue (Supplementary Fig. 1j–m).

To investigate the role of NF2 in UB branching, we deleted *Nf2* within the UB lineage using *Hoxb7:Cre* (ref. 19). *Hoxb7:Cre*^*tg/+*^ *Nf2*^*flox/flox*^ (*Nf2*^*UB−/−*^) newborns (P0) were obtained at Mendelian ratios; however, only 6% of *Nf2*^*UB−/−*^ pups survived to weaning suggesting early postnatal lethality. *Nf2*^*UB−/−*^ newborns had severe kidney hypodysplasia (kidney area in *Nf2*^*UB−/−*^: 1.1±0.7 mm^2^, *n*=14; controls: 4.8±0.5 mm^2^, *n*=18, Fig. 1b–e). Histological examination of P0 kidneys revealed cortex-medulla disorganization and dilated tubules with limited number of glomeruli (Fig. 1f–h, Supplementary Fig. 1z,za). Staining for HNF4α (proximal tubule marker), and CALBINDIN (collecting duct and distal tubule marker) revealed dilations were both autonomous and non-cell-autonomous (Supplementary Fig. 1n–q). In rare cases where *Nf2*^*UB−/−*^ animals survived to weaning, kidneys were smaller with tubule dilations and parenchyma destruction (Supplementary Fig. 1r,s).

### NF2 is required for branching morphogenesis

**Figure 2:**
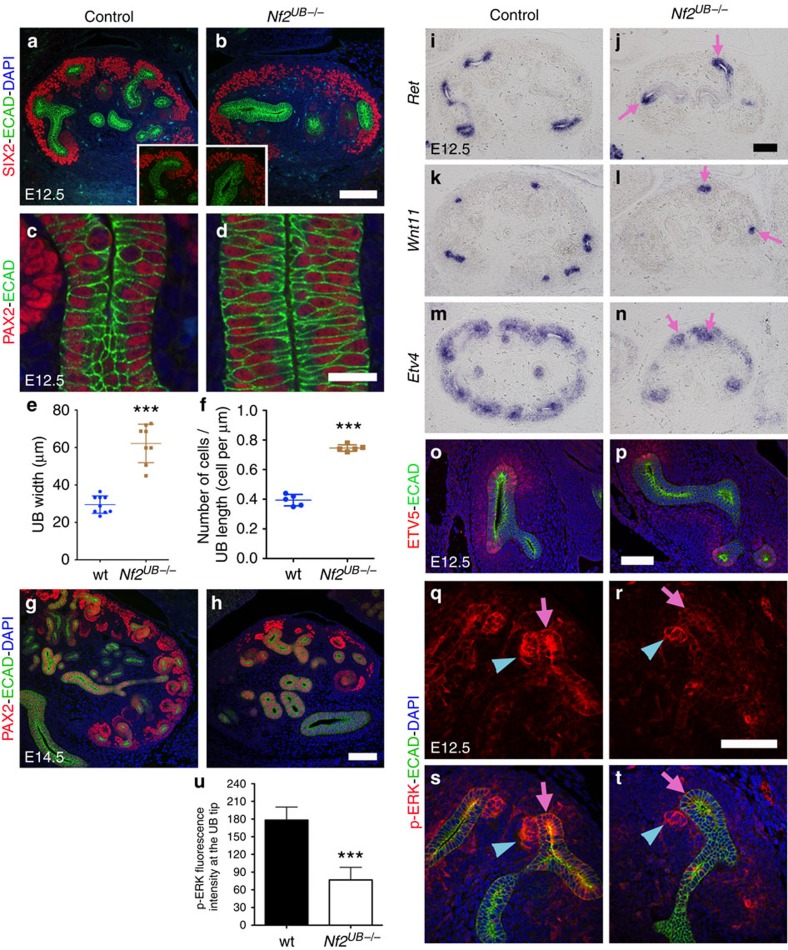
*Nf2* deletion leads to pseudostratification of the ureteric epithelium and reduced MAPK signalling (**a**,**b**) Loss of *Nf2* leads to reduced UB branching, with sparse and reduced CM population at E12.5. (**c**,**d**) PAX2/E-CADHERIN staining reveals increased UB width and cellularity in *Nf2*^*UB−/−*^ mutants compared with controls at E12.5. (**e**,**f**) Quantification at E12.5 reveals a 2.2 and 1.85-fold increase in UB width and cellularity, respectively, in *Nf2*^*UB−/−*^ compared with controls. (**g**,**h**) In *Nf2*^*UB−/−*^ kidneys, loss of CM cell population (PAX2) was apparent at E14.5. (**i**–**p**) UB tip-specific markers (*in situ* hybridizations: *Ret*, *Wnt11*, *Etv4*, immunostaining: ETV5) are expressed in the few UB tips that form in *Nf2* mutants. (**q**–**t**) Phospho-ERK staining is greatly reduced in *Nf2*^*UB−/−*^ UB tips compared with controls. Pink arrows point to UB tips, while blue arrowheads point to early nephron structures. (**u**) Quantification of p-ERK signal at the UB tips using five kidneys per genotype. Error bars represent s.d., ***P<0.0001, Student's *t*-test. Scale bars represent 100 μm (**a**,**b**, **g**–**t**) and 20 μm (**c**,**d**).

To examine the role of NF2 in ureter branching in greater detail, we placed T-stage (2 UB tips) kidney rudiments dissected from wild-type and *Nf2*^*UB−/−*^ E11.5 embryos in culture and counted the number of UB tips after 48 h. Strikingly, deletion of *Nf2* resulted in loss of ureter branching (number of UB tips: 2±0.4, *n*=21) compared with controls (23.5±4.7, *n*=21, Fig. 1i–k). Interestingly, while NF2 function is dispensable for the formation of the UB (marked by PAX2) and its first branching event (Fig. 1l,m), subsequent branching failed in the absence of *Nf2* (Fig. 1n–q and 2a,b).

Despite the loss of branching, *Nf2*-depleted UBs continued to grow, as indicated by the wider epithelial tubules observed in *Nf2*^*UB−/−*^ kidneys compared with controls (Fig. 1i,j and 2c,d). Quantification at E12.5 revealed a 2.2-fold increase in UB width and 1.85-fold increase in cellularity in the UB compartment of *Nf2*^*UB−/−*^ embryos compared with controls (Fig. 2e,f). At E12.5, the dense packing leads to the appearance of stratification; however, confocal z-stack imaging of PAX2/E-CADHERIN staining revealed that UB cells in *Nf2* mutants contact both apical and basal surfaces, consistent with a pseudostratified structure (Supplementary Movie 1 (control) and Supplementary Movie 2 (*Nf2*^*UB−/−*^)).

To ascertain if altered apoptosis could explain the morphological defects seen in *Nf2* mutants, we examined CLEAVED CASPASE 3 (CC3) staining in control and *Nf2*^*UB−/−*^ kidneys at E12.5. CC3-positive cells were rarely detectable in *Nf2*^*UB−/−*^ and control UB cells (marked by CALBINDIN) suggesting cell death is not the primary cause of defective branching (Supplementary Fig. 1t,u). Quantification revealed higher apoptotic rates in the cortex of *Nf2*^*UB−/−*^ compared with controls (Supplementary Fig. 1v) consistent with known roles of branching in sustaining survival of the mesenchyme. Quantification of EdU incorporation at E11.5 showed no change in proliferation of UB cells (Supplementary Fig. 1w–y). We conclude that neither proliferation nor apoptosis are primarily responsible for *Nf2*^*UB−/−*^ branching defects.

Branching morphogenesis during kidney development involves signalling between the UB, metanephric mesenchyme and stromal compartments^1^,^3^. PBX1 staining revealed normal differentiation of the stromal compartment in *Nf2* mutants (Supplementary Fig. 2c,d). Staining with condensing mesenchyme (CM) markers (SALL1, SIX2 and PAX2) revealed a decreased CM population at E12.5 and E13.5, and a depletion of CM at E18.5 in *Nf2*^*UB−/−*^ kidneys (Fig. 2a,b,g,h and Supplementary Fig. 2a–h). PAX2 staining on kidney explants confirmed the decreased CM population in *Nf2* mutants compared with controls (Supplementary Fig. 2i,j). Moreover, we observed a difference in the morphology of the CM domain, as SIX2 cells appeared sparser in *Nf2*^*UB−/−*^ kidneys compared with controls (compare SIX2 staining in Fig. 2a,b and Supplementary Fig. 2e,f).

### NF2 is critical for RET-ERK signalling at UB tips

GDNF/RET signalling plays an essential role in UB branching^5^,^6^,^7^. GDNF binding to its receptors RET and GFRα1 induces both ERK/MAPK and PI3K pathway activation. PI3K activation is critical for UB branching, and necessary for activation of downstream RET target genes ETV4 and ETV5 (ref. 20). Pharmacological inhibition of the ERK/MAPK pathway or loss of *Mek1/2* also results in impaired branching but without affecting the expression of ETV4/5 (ref. 21). Thus both ERK/MAPK and PI3K signalling contribute to branching morphogenesis.

To test if the RET pathway is affected by *Nf2* deletion, we performed *in situ* hybridization and immunostaining for *Ret* and its downstream targets. Although *Nf2*^*UB−/−*^ kidneys had fewer UB tips compared with controls, *Ret* was expressed at levels comparable to controls at UB tips (Fig. 2i,j). We next examined RET signalling activity. No changes in *Etv4*, ETV5 and phospho-AKT were observed in *Nf2*^*UB−/−*^ kidneys compared with controls (Fig. 2m–p, Supplementary Fig. 2s–v) suggesting that NF2 does not primarily affect PI3K activation. Interestingly, expression of tip marker genes (*Wnt11*, *Crlf1*, *Sprouty1* and *Dusp6)* are seen at the few tips remaining in *Nf2*^*UB−/−*^ kidneys (Fig. 2k,l, and Supplementary Fig. 2m–r). We next examined ERK/MAPK pathway activation with antibodies to p-ERK (Fig. 2q–u). In controls, p-ERK staining was observed at the UB tips and in early nephrons. In *Nf2*^*UB−/−*^ mutants, p-ERK staining persisted in early nephrons, but UB tip staining was greatly reduced suggesting that loss of *Nf2* may impair UB branching due to defective ERK/MAPK activation.

### *Yap*/*Taz* haploinsufficiency rescues the *Nf2* ^*UB−/−*^ phenotype

Since NF2 is required for tight junction formation and cell polarity in the epidermis^22^, we examined *Nf2*^*UB−/−*^ kidneys for CRUMBS3, ZO-1, β-CATENIN and LAMININ. All these markers revealed that UB cells depleted for *Nf2* had normal apico-basal polarity at the time of defective branching (Supplementary Fig. 3a–f).

**Figure 3:**
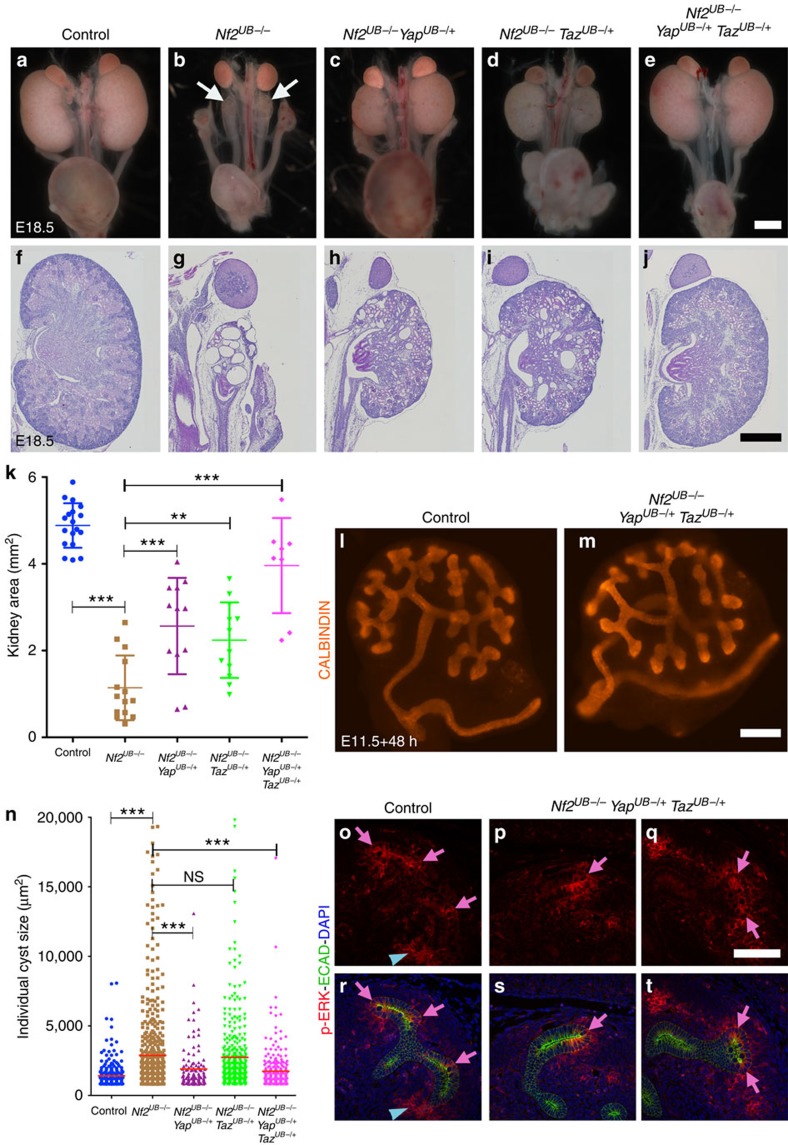
*Yap* and *Taz* haploinsufficiency suppresses *Nf2*^*UB−/−*^ kidney hypodysplasia. Macroscopic (**a**–**e**) and periodic acid-Schiff (PAS) staining (**f**–**j**) of E18.5 kidneys reveals extent of rescue of the *Nf2*^*UB−/−*^ phenotype by *Yap* and/or *Taz* haploinsufficiency. (**k**) Quantification of kidney sizes in different genotypes (control (*n*=18 kidneys), *Nf2*^*UB−/−*^ (*n*=14), *Nf2*^*UB−/−*^ *Yap*^*UB−/+*^ (*n*=12), *Nf2*^*UB−/−*^ *Taz*^*UB−/+*^ (*n*=12) and *Nf2*^*UB−/−*^ *Yap*^*UB−/+*^ *Taz*^*UB−/+*^ (*n*=8). Error bars represent s.d., **P<0.005 and ***P<0.0001, Student's *t*-test). (**l**,**m**) Reduction of *Yap* and *Taz* levels in *Nf2*^*UB−/−*^ mutants rescues branching in kidney explants. (**n**) Quantification of cyst sizes in different genotypes. (Control (*n*=271 cysts), *Nf2*^*UB−/−*^ (*n*=662), *Nf2*^*UB−/−*^ *Yap*^*UB−/+*^ (*n*=126), *Nf2*^*UB−/−*^ *Taz*^*UB−/+*^ (*n*=535) and *Nf2*^*UB−/−*^ *Yap*^*UB−/+*^ *Taz*^*UB−/+*^ (*n*=316). Error bars represent s.d., NS, not significant, ***P<0.0001, Student's *t*-test). (**o**–**t**) Phospho-ERK expression is rescued in *Nf2*^*UB−/−*^ *Yap*^*UB−/+*^ *Taz*^*UB−/+*^ UB tips to control levels. Pink arrows point to UB tips, while blue arrowheads point to early nephrons. Scale bars represent 1 mm (**a**–**e**), 0.5 mm (**f**–**j**), 200 μm (**l**,**m**) and 100 μm (**o**–**t**).

*Nf2* deletion can inhibit the Hippo pathway, leading to reduced YAP/TAZ phosphorylation and increased nuclear YAP/TAZ (refs 16, 23). We did not observe any changes in levels, phosphorylation or subcellular localization of YAP and TAZ in *Nf2*^*UB−/−*^ compared with control kidneys at E12 (Supplementary Fig. 3g–r). No significant changes were detected in western blot analysis using YAP, p-YAP, MST, p-MST, LATS and p-LATS antibodies in E13.5 *Nf2*^*UB−/−*^ and control kidneys (Supplementary Fig. 3s). However, it is possible that we failed to detect subtle changes in YAP levels that nonetheless were functionally relevant. Therefore, we decided to genetically test whether excess YAP/TAZ activity in *Nf2* mutants inhibits UB branching. We attempted to rescue the *Nf2*^*UB−/−*^ phenotype by reducing YAP and TAZ levels, by generating *Hoxb7:Cre*^*tg/+*^ *Nf2*^*flox/flox*^ *Yap*^*flox/+*^ *Taz*^*flox/+*^ embryos (*Nf2*^*UB−/−*^ *Yap*^*UB−/+*^ *Taz*^*UB−/+*^). While E18.5 *Nf2*^*UB−/−*^ mice had severe kidney hypodysplasia (*Nf2*^*UB−/−*^: 1.1±0.7 mm^2^), removing one allele of each *Yap* and *Taz* in *Nf2*^*UB−/−*^ embryos remarkably restored kidney size (*Nf2*^*UB−/−*^ *Yap*^*UB−/+*^*Taz*^*UB−/+*^: 3.9±1.1 mm^2^) almost to wild-type size (4.8±0.5 mm^2^). Removal of either one copy of *Yap* (*Nf2*^*UB−/−*^ *Yap*^*UB−/+*^: 2.5±1.1 mm^2^) or one copy of *Taz* (*Nf2*^*UB−/−*^ *Taz*^*UB−/+*^: 2.2±1.1 mm^2^) partially rescued the phenotype (Fig. 3a–k).

To assess branching morphogenesis in greater detail, we dissected wild-type and *Nf2*^*UB−/−*^ *Yap*^*UB−/+*^ *Taz*^*UB−/+*^ kidney rudiments at E11.5 and placed them in culture for 48 h. Lowering YAP/TAZ dosage in *Nf2* mutants largely rescued the branching capacity of kidney explants (Fig. 3l,m). Reducing YAP and TAZ also decreased the non-cell-autonomous tubule dilatation (Fig. 3n), and restored phospho-ERK expression (Fig. 3o–t). Remarkably, while most of *Nf2* mutants died within 48 h after birth, all *Nf2*^*UB−/−*^ *Yap*^*UB−/+*^ *Taz*^*UB−/+*^ mice lived to adulthood (*n*=5 animals). These results suggest that the loss of branching morphogenesis in *Nf2* mutants is due to excessive YAP/TAZ activity.

### YAP expression reversibly inhibits branching

Our rescue experiments suggest that increased YAP/TAZ activities in *Nf2*^*UB−/−*^ inhibit UB branching. To directly test this hypothesis, we overexpressed YAP in the UB lineage by generating *Hoxb7:Cre*^*tg/+*^ *Rosa26-lox-STOP-lox-rtta-IRES-EGFP Yap*^*Tg*^ animals (called *Yap*^*UB-OE*^). This approach allows constitutive expression of the wild type, HA-tagged form of YAP in the UB compartment after doxycycline treatment either by feeding pregnant dams with doxycycline food or by adding doxycycline to the kidney culture medium.

**Figure 4:**
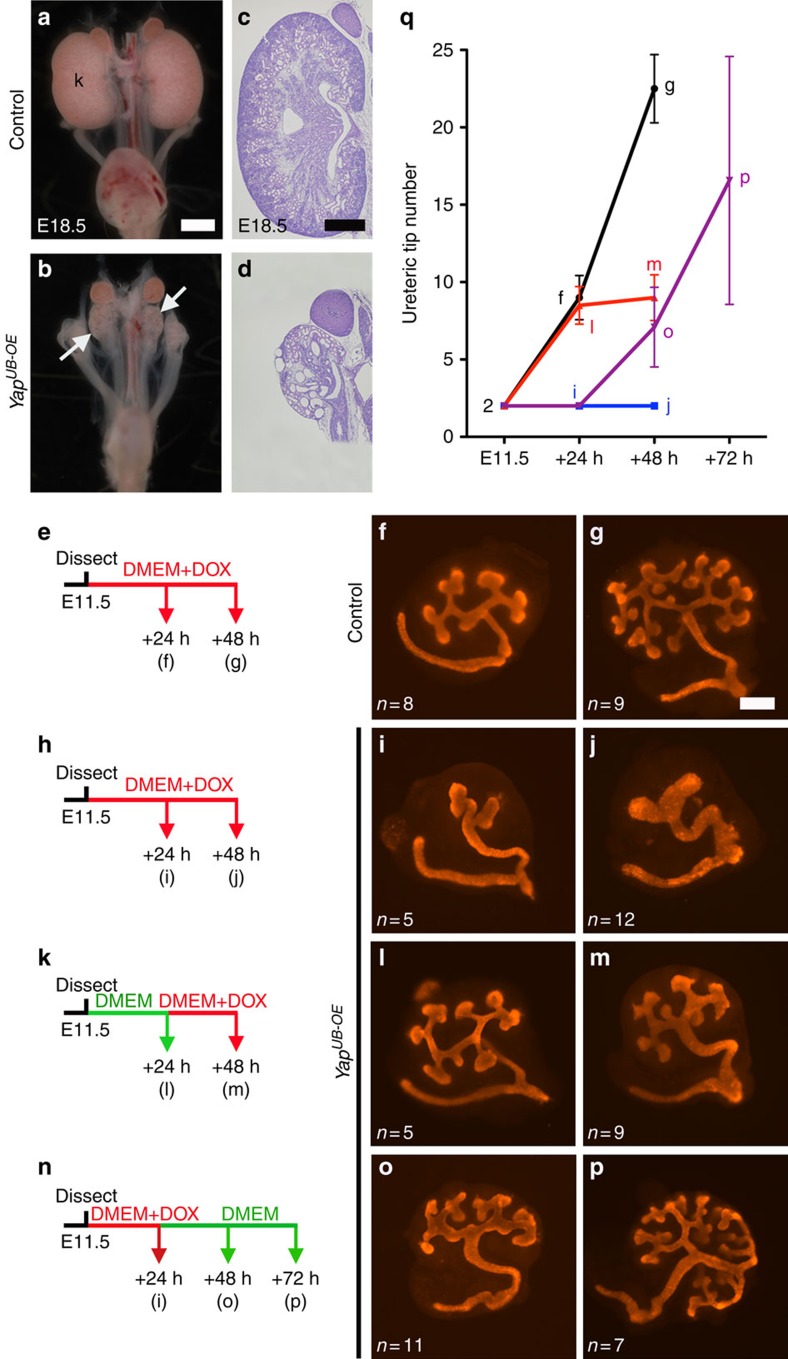
YAP overexpression in the UB epithelium blocks branching. (**a**–**d**) Macroscopic view and periodic acid-Schiff (PAS) staining of YAP overexpressing and control kidneys at E18.5. Pregnant dams have been fed with doxycycline food (0.645 g kg^−1^) from E11 to E18.5. (**e**–**p**) E11.5 T-stage control kidney explants cultured *ex vivo* in presence of 1,500 ng ml^−1^ of doxycycline for 24 and 48 h shows the stereotypical branching morphogenesis pattern with an average of 22 ureteric tips in control kidneys after 48 h of cultures (**e**–**g**). (**h**–**j**) No branching is observed in *Yap*^*UB-OE*^ kidneys as no new tip forms within 2 days in culture. (**k**–**m**) After 24 h of culture in normal media, the addition of doxycycline and induction of YAP transgene expression results in a complete block of further branching. (**n**–**p**) Branching morphogenesis blockage requires continuous YAP overexpression, as withdrawal of doxycycline from the medium after 24 h releases the inhibition, and new tips are formed. (**q**) Quantification of UB tip number in *ex vivo* explants experiments. Panels **e**,**h**,**k** and **n** represent the experimental flow of the *ex vivo* kidney cultures. The number of explants analysed for each genotype is indicated in the lower left corner of each panel. Scale bars represent 1 mm (**a**,**b**), 0.5 mm (**c**,**d**) and 250 μm (**f**–**p**).

Initially, we overexpressed YAP in the UB lineage throughout kidney development by feeding pregnant dams with doxycycline (0.625 g kg^−1^) from E11 to E18.5. *Yap*^*UB-OE*^ animals developed severe renal hypodysplasia at E18.5 (Fig. 4a–d). To explore if YAP overexpression in the UB lineage affected branching, we cultured E11.5 *Yap*^*UB-OE*^ kidney rudiments (T-stage) in presence of doxycycline (1,500 ng ml^−1^), and counted the number of UB tips 24 and 48 h later. Interestingly, while control kidneys had 9±2.8 and 22.5±1.3 UB tips after 24 and 48 h, respectively (Fig. 4e–g,q), doxycycline-induced YAP overexpression in the UB led to a complete blockage of branching (2±0 UB tips, Fig. 4h–j,q and Supplementary Fig. 4a). Similarly, UB branching was blocked when doxycycline was added to the medium 24 h after dissection (Fig. 4k–m,q). Remarkably, when doxycycline was withdrawn from the medium, UB branching resumed (Fig. 4n–p,q). Thus, YAP rapidly and effectively blocks branching in a reversible manner.

**Figure 5:**
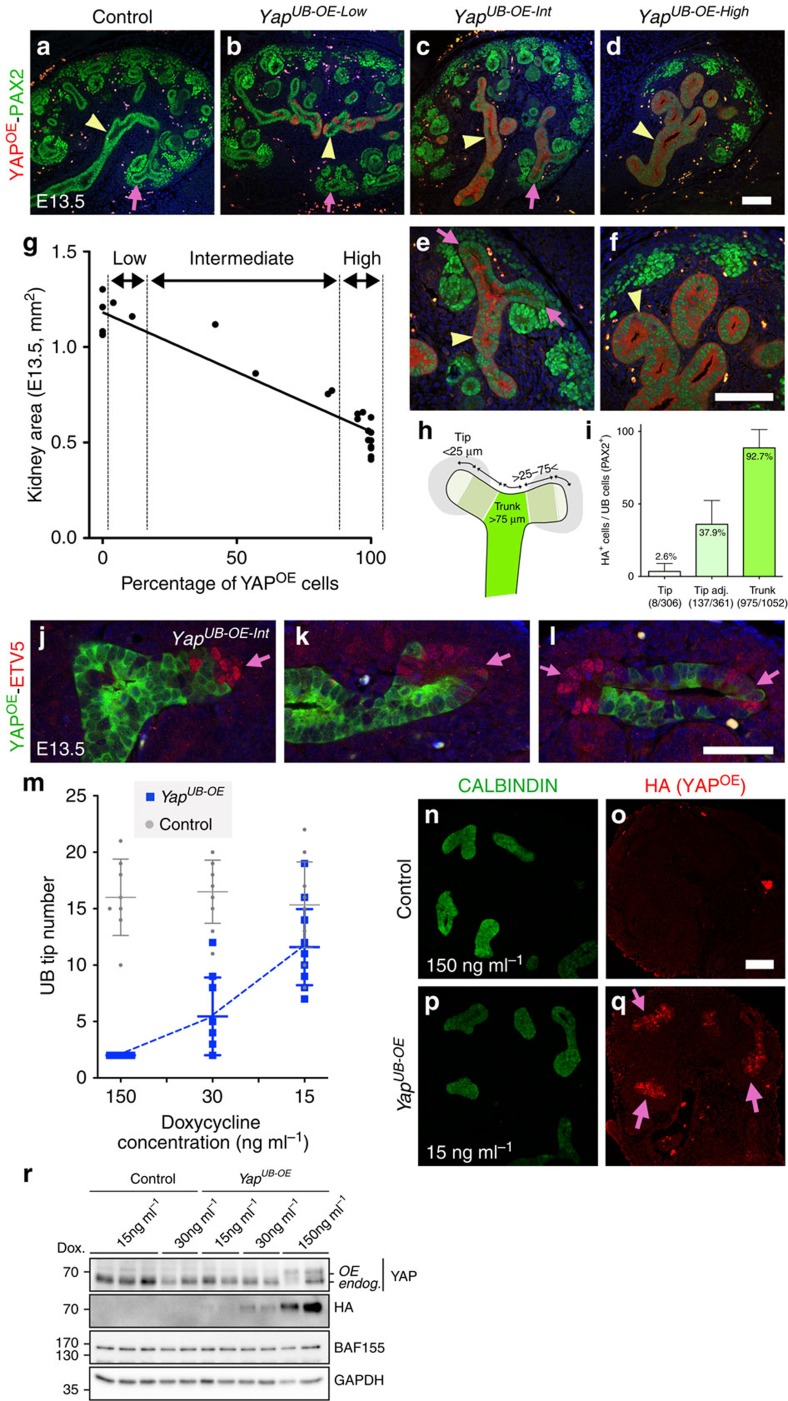
Mosaic analysis reveals YAP overexpressing cells rarely contribute to UB tips. (**a**–**l**) Pregnant dams were fed with doxycycline food (0.645 g kg^−1^) from E11 until E13.5. YAP^OE^ cells (red – HA positives) are only detected in triple transgenic (*Hoxb7:Cre*^*tg/+*^ *Rosa26-lox-STOP-lox-rtta-IRES-EGFP Yap*^*Tg*^) mice (**b**–**f**) and not in control animals (**a**). (**e**,**f**) Higher magnification views of **c** and **d**. (**g**) Quantification reveals that the percentage of YAP^OE^ cells in the UB epithelium correlates with kidney size. (**h**) Diagram of denomination of tip (within 25 μm from the tip), tip adjacent (cells located 25–75 μm away from the tip) and trunk (cells located 75 μm and more from the tip) domains. (**i**) Quantification of the distribution of YAP^OE^ cells in the UB compartments (tip, tip adjacent and trunk) in *Yap*^*UB-OE-Int*^ mutants. Numbers in brackets represent the number of HA-positive cells and the total number of counted cells. (**j**–**l**) Immunostaining using anti-HA and ETV5 antibodies shows that the rare YAP^OE^ cells present in the tip domain do not expressed the UB tip marker ETV5. Separate channels are shown in Supplementary Fig. 5h–m. (**m**) Quantification of UB tip numbers of E11.5 control and *Yap*^*UB-OE*^ kidneys, after 48 h in culture exposed to different concentrations of doxycycline (15, 30 and 150 ng ml^−1^). Quantification was made on 8, 10 and 12 control explants and 7, 10 and 12 *Yap*^*UB-OE*^ explants at 150, 30 and 15 ng ml^−1^, respectively. (**n**–**q**) HA staining on kidney explant sections treated with 15 ng ml^−1^ of doxycycline (low enough to allow branching) reveals that cells with low YAP overexpression can contribute to UB tips. (**r**) Western blot analysis of kidney lysates confirms activation of YAP expression at different concentrations of doxycycline used in panels **m**–**q**. Scale bars represent 100 μm (**a**–**f**, **n**–**q**) and 50 μm (**j**–**l**).

Constitutive YAP overexpression resulted in UB branching arrest (Fig. 4), but UBs overexpressing YAP continued to grow, as indicated by the wider epithelial tubules observed in *Yap*^*UB-OE*^ kidneys explants (Fig. 4i,j). Similarly, feeding pregnant dams with doxycycline diet from E11 to E13.5 was sufficient to activate YAP expression (Supplementary Fig. 5o,q), block UB branching, and led to increased UB width and epithelial pseudostratification in E13.5 *Yap*^*UB-OE*^ embryos (Fig. 5d,f). As with *Nf2* mutants, proliferation in *Yap*^*UB-OE*^ mutants was unaffected at E13.5 (Supplementary Fig. 5e–g). Loss of *Nf2* or YAP overexpression blocked branching and resulted in a pseudostratified epithelium. We speculate this is due to the continuation of proliferation in the absence of branching. Thus, increased YAP activity in the UB lineage leads to loss of branching.

### YAP overexpressing cells are excluded from UB tips

YAP overexpression throughout the UB lineage inhibits branching morphogenesis and leads to kidney hypoplasia (Fig. 4). Interestingly, due to the occasional mosaic activity of the *Hoxb7:Cre* line, we observed that kidney hypoplasia correlated with the percentage of YAP overexpressing (YAP^OE^) cells (Fig. 5a–g). We arbitrary divided the *Yap*^*UB-OE*^ animals in three categories (low, intermediate and high) based on the percentage of YAP^OE^ cells in the ureteric epithelium. Kidneys with a low percentage of YAP^OE^ cells (<12%), were similar in size to controls, while those with a high percentage (> 86%) were severely reduced in size, likely as a consequence of branching arrest (Fig. 5g).

Additionally, we took advantage of the mosaicism to analyse the contribution of YAP^OE^ cells to UB tips and trunks, compared with wild-type cells. We used an anti-HA antibody to identify cells that overexpressed transgenic YAP (HA-YAP), and PAX2 to label the entire ureteric tree. Remarkably, cells that overexpressed YAP rarely contributed to the UB tips (2.6%), and their distribution increased away from the tips (37.9% in tip adjacent domain), with nearly all trunk cells being HA positive (92.7%, Fig. 5c,e,i and Supplementary Fig. 5a,b). Even in low YAP overexpressing kidneys (*Yap*^*UB-OE-Low*^), YAP^OE^ cells were not randomly distributed, but exclusively found in the trunk (Fig. 5b, and Supplementary Fig. 5c,d). Importantly, in rare cases where HA cells did contribute to UB tips (2.6%), expression of ETV5 was not detected (Fig. 5j–l and Supplementary Fig. 5h–m).

Next, we wondered if the tip exclusion behaviour of YAP^OE^ cells is linked to the level of YAP overexpression. We first examined the impact of increasing doxycycline exposition (pregnant dams fed with 0.2, 0.645 (the same concentration used above) and 2 g kg^−1^ doxycycline food from E11 to E18.5) on kidney branching. Remarkably, all diets led to severe kidney hypoplasia (Supplementary Fig. 5n), likely due to the high level of YAP overexpression observed even at low dose (Supplementary Fig. 5o–r). Therefore, to better control the level of YAP overexpression, we examined the effect of varying the concentrations of doxycycline in cultured kidneys and analysed YAP induction after 24 h using western blot analysis. We also quantified UB branching after 48 h using CALBINDIN staining. Decreasing the level of doxycycline to 15 ng ml^−1^ resulted in branching similar to controls (Fig. 5m), with HA-positive cells observed in the entire UB compartment (UB tip and trunk, as observed from kidney explant sections in Fig. 5n–q). Thus cells with low levels of YAP overexpression (Fig. 5r) can contribute to active UB tips. We conclude that cells with high levels of YAP activity have a cell-autonomous defect in their ability to contribute to the UB tip domain and RET signalling.

### LATS is required for branching via inhibition of YAP and TAZ

**Figure 6:**
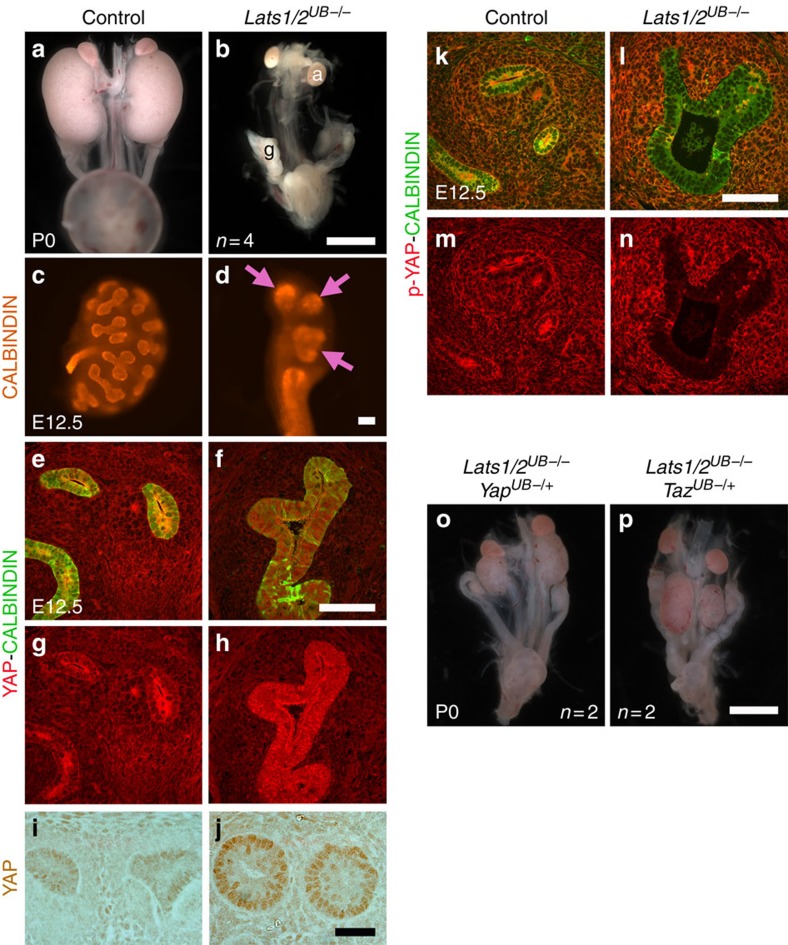
The Hippo kinases LATS1 and LATS2 control branching through YAP/TAZ. (**a**,**b**) Macroscopic views of the urogenital system from controls and *Lats1/2*^*UB−/−*^ mutants at P0. Note the bilateral kidney agenesis in double *Lats1/2*^*UB−/−*^ embryos. a, adrenal; g, gonads. (**c**,**d**) Branching morphogenesis at E12.5 is severely reduced in *Lats1/2*^*UB−/−*^ embryos compared with controls as seen with whole-mount CALBINDIN immunostaining. Pink arrows point to the UB tips. (**e**–**n**) *Lats1/2* deletion causes increased nuclear YAP (**e**–**j**), and decreased phospho-YAP (**k**–**n**) levels in the UB, marked by CALBINDIN. (**o**,**p**) *Yap* and *Taz* heterozygosity rescues the *Lats1/2*^*UB−/−*^ kidney agenesis phenotype as kidneys form in *Lats1/2*^*UB−/−*^ *Yap*^*UB−/+*^ and *Lats1/2*^*UB−/−*^ *Taz*^*UB−/+*^ embryos. Scale bars represent 1 mm (**a**,**b**,**o**,**p**), 100 μm (**c**–**h**,**k**–**n**) and 50 μm (**i**,**j**).

To test whether the core Hippo kinases LATS1 and LATS2 are essential for branching morphogenesis, we examined the consequence of removing *Lats1* and *Lats2* from the UB lineage (*Hoxb7:Cre*^*tg/+*^ *Lats1*^*flox/flox*^ *Lats2*^*flox/flox*^, called *Lats1/2*^*UB−/−*^). Normal kidney development was observed even in the absence of three out of four *Lats1* and *Lats2* alleles, regardless of allelic combinations (Supplementary Fig. 6a–c). However, double *Lats1* and *Lats2* conditional knock-out embryos showed bilateral kidney agenesis at P0 (no kidneys observed in all four *Lats1/2*^*UB−/−*^ embryos, Fig. 6a,b). Earlier examination revealed that the UB formed, but subsequent branching morphogenesis was severely disrupted as seen by the reduced number of UB tips at E12.5 (control: 15±3.5; *Lats1/2*^*UB−/−*^: 2±0.8, Fig. 6c,d). Immunostaining of *Lats1/2*^*UB−/−*^ kidneys at E12.5 showed increased nuclear YAP protein levels accompanied by a loss of phospho-YAP in the UB compartment compared with controls (Fig. 6e–n). Importantly, introducing *Yap* or *Taz* haploinsufficiency into *Lats1/2* mutants rescued kidney agenesis observed at birth (*Lats1/2*^*UB−/−*^ *Yap*^*UB−/+*^=4 kidneys in 2 embryos, *Lats1/2*^*UB−/−*^ *Yap*^*UB−/+*^=4 kidneys in 2 embryos, Fig. 6o,p). Interestingly, *Lats1/2* deletion resulted in increased CRUMBS3 and E-CADHERIN apical staining, and abnormal localization of E-CADHERIN to the basal membrane (Supplementary Fig. 6d–i). Thus, the core Hippo kinases LATS1/2 are essential in the UB lineage to maintain cell polarity, restrict YAP/TAZ activity and to allow branching morphogenesis.

### *Yap/Taz* deletion expands UB tip leading to dilated branching

**Figure 7:**
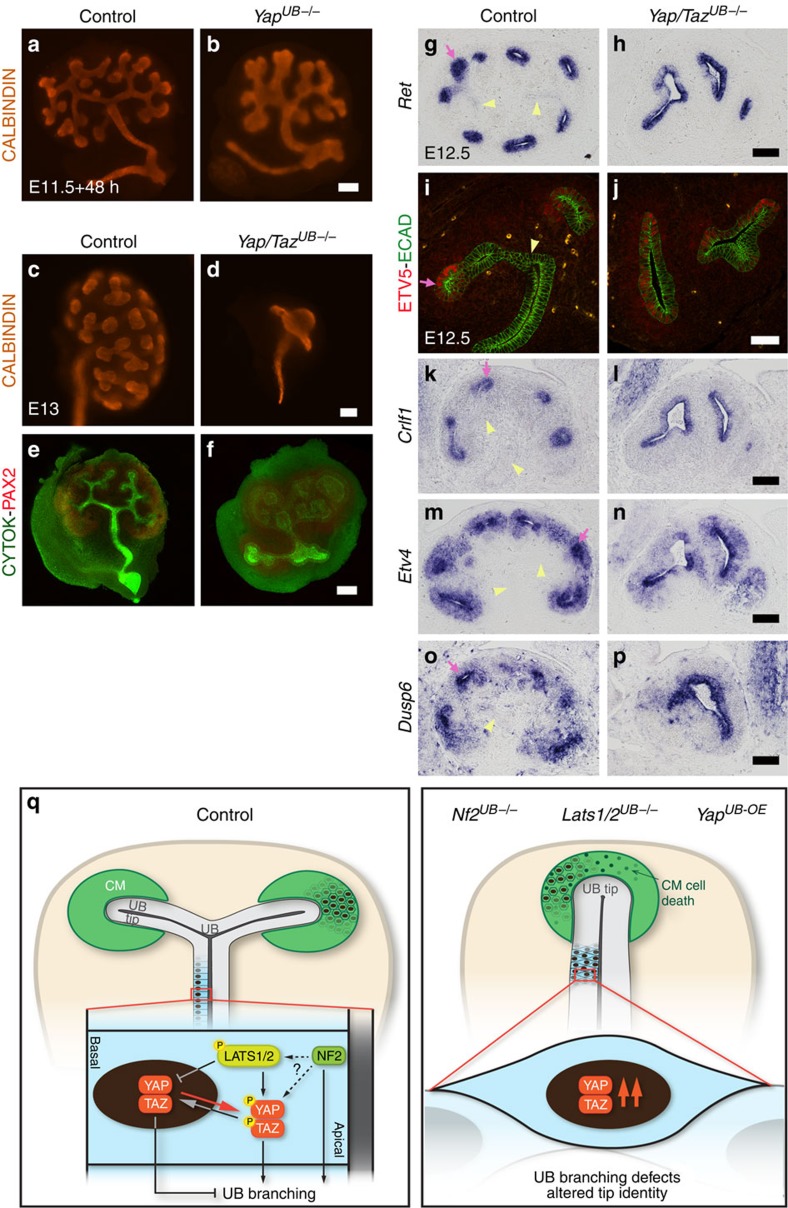
YAP and TAZ control UB tip fate and structure. (**a**,**b**) Kidney explants of *Yap*^*UB−/−*^ mutants show reduced branching with cyst-like morphology after 48 h. (**c**–**f**) Whole-mount immunostaining reveals abnormal UB branching of *Yap/Taz*^*UB−/−*^ embryos compared with controls *in vivo* (E13, **c**,**d**) and *ex vivo* (kidney culture, **e**, **f**). (**g**–**p**) In E12.5 controls, *Ret*, ETV5, *Crlf1*, *Etv4* and *Dusp6* are expressed in UB tips (pink arrows) but not in trunk segments (yellow arrowheads). In *Yap/Taz*^*UB−/−*^ mutants, the entire UB epithelium expresses these genes, suggesting a shift towards tip identity. (**q**) Model figure: see discussion for description. Scale bars represent 100 μm.

Our data clearly show that high YAP/TAZ activity is incompatible with UB branching morphogenesis. To determine if *Yap* and *Taz* expression is essential for branching morphogenesis, we examined *Yap* and *Taz* mutants using the *Hoxb7:Cre*. Dilated tip structures were apparent in *Yap*^*UB−/−*^ kidney explants (Fig. 7a,b), while UB depleted of *Taz* branched normally (data not shown). Double *Yap/Taz*^*UB−/−*^ kidneys were hypoplastic with decreased proliferation (Supplementary Fig. 7a–c) and severe disruptions in branching morphogenesis, resulting in cyst-like branching (Fig. 7c–f). In controls, expression of Ret and its downstream targets ETV5, *Crlf1*, *Etv4* and *Dusp6* were restricted to the UB tips, whereas in *Yap/Taz* mutants, their expression domains expanded (Fig. 7g−p). This suggests that in the absence of *Yap* and *Taz*, UB tip fate expands. Thus *Yap/Taz* deletion from the UB epithelium leads to the opposite effect from loss of *Nf2* and excess YAP, namely expanded expression of RET pathway/tip genes and cyst-like branching, that may represent unrestricted outward expansion of a tip fate.

## Discussion

We have uncovered an unexpected role for NF2 and the Hippo pathway in regulating branching morphogenesis in the mammalian kidney. We found that NF2 and LATS1/2 are required in the UB lineage to restrain YAP/TAZ activities, and showed that YAP overexpression is sufficient to block branching morphogenesis. In addition, reducing YAP and TAZ levels can rescue branching defects of both *Lats1/2* and *Nf2* mutants. This suggests that increased YAP/TAZ activity is the primary cause of defective branching on loss of *Nf2* and *Lats1/2*.

Using mosaic analysis, we also found that cells overexpressing YAP have a cell-autonomous defect in their ability to contribute to the UB tip domain. Thus, cells with heightened YAP levels, similar to *Ret*^*−/−*^ and *Etv5*^*−/−*^ cells^3^,^24^, are excluded from tips. Strikingly, rare YAP^OE^ cells found at tips failed to activate RET signalling in a cell-autonomous manner. In contrast, UB depleted for *Yap* and *Taz* showed expansion of the tip domain. Taken together, these data suggest YAP/TAZ inhibits tip fate and signalling.

We cannot distinguish if YAP directly represses RET signalling or represses tip fate, thereby affecting RET signalling. Either scenario might allow a splitting of the tip, allowing new branch formation. How might this occur? One attractive scenario for the action of YAP/TAZ in symmetry breaking is that crowding that occurs at tips^25^, impacts the levels of nuclear YAP/TAZ and thereby RET signalling. This would split the tip domain, leading to branch formation. The positive feedback of RET signalling to increased *Ret* transcription would then promote further growth of each tip, until a critical point of cell crowding and tension occurs at a new ampulla, which would then again regulate nuclear YAP, causing splitting of the ampulla and branch formation.

Tip cells produce secreted paracrine signals that maintain and pattern both the stromal and mesenchymal compartments. Interestingly, *Nf2*^*UB−/−*^ and *Yap*^*UB-OE*^ kidneys showed rapid depletion of the CM (as early as E13.5) suggesting that increased YAP activity at the tips is detrimental to the expression of an unidentified signal that maintains the CM. We noted cell death in the *Nf2*^*UB−/−*^ outer cortex, likely due to the fact that CM cells receive reduced inductive signals from the smaller number of UB branches that formed.

*Nf2* deletion leads to both cell-autonomous and non-cell-autonomous tubule dilations at P0. The non-cell-autonomous effects may be due to secondary defects subsequent to loss of renal structure. Interestingly, lowering YAP and TAZ levels in the UB compartment of *Nf2* mutants significantly reduced tubule dilatations suggesting that when NF2 inhibits YAP and TAZ in the UB compartment to promote branching morphogenesis, it may also restore inductive signals to the adjacent CM for nephron formation. It is interesting to note that while *Yap* or *Taz* haploinsufficiency partially rescues the branching defect of *Nf2* mutants, *Taz* haploinsufficiency has only a limited ability to rescue tubule dilatations compared with *Yap* haploinsufficiency. This confirms that YAP and TAZ have redundant function in branching^26^, and suggests that YAP and TAZ have different functions in restricting tubule dilations.

If both NF2 and LATS1/2 inhibit YAP/TAZ activity, why does *Lats1/2* UB-deletion give rise to stronger phenotype than *Nf2* deletion? Phospho-YAP staining persists in *Nf2*^*UB−/−*^, while it is absent in *Lats1/2*^*UB−/−*^ kidneys, suggesting that in the absence of *Nf2*, LATS1 and LATS2 kinases are still active. Our study suggests that LATS1/2 kinases might also have other roles than YAP/TAZ phosphorylation. We found that *Lats1/2*^*UB−/−*^ displayed obvious polarity defects, not observed in *Nf2*^*UB−/−*^ or *Yap*^*UB-OE*^ (Supplementary Fig. 4b–g). The suppression of defects of both *Lats1/2* and *Nf2* mutants by reducing YAP/TAZ dosage is consistent with a linear NF2-LATS1/2-YAP/TAZ activity pathway, but does not exclude other models (Fig. 7q).

While our genetic analysis clearly demonstrates an essential role for tight regulation of YAP/TAZ activity during branch formation, we could not detect a clear difference in the localization of YAP in the tips compared with the trunk. We speculate that changes in YAP/TAZ localization are transient and/or only occur at low levels. We note that changes in Yorkie localization are not detectable in *Drosophila* discs in normally proliferating cells, suggesting that very low levels of YAP, undetectable by staining, can still be potent in transcriptional regulation. *In vivo* reporters of YAP/TAZ activity that can be imaged at real-time in the mouse kidney will be needed to obtain a better resolution of changes that are occurring at tips during development.

In conclusion, we have shown that NF2, LATS1/2, YAP and TAZ have critical roles in branching morphogenesis in the mammalian kidney. We propose a model in which the environment at the tip alters YAP/TAZ activity, which in turn feeds back to inhibit tip fate/signalling, break symmetry at the ampulla and promote branch formation. Since branching morphogenesis is critical for the formation of many organs, we speculate that the Hippo pathway will also play critical roles in branch formation in other tissues.

## Methods

### Mouse lines

*Hoxb7:Cre*^*tg/+*^, *Taz*^*flox*^ and *Yap*^*flox*^*, Lats1*^*flox*^, *Lats2*^*flox*^, *Nf2*^*flox*^ and *Yap*^*Tg*^ mouse strains were generously provided and have been described elsewhere^19^,^27^,^28^,^29^,^30^. All mice were maintained in a mixed genetic background and both male and female mice were assessed in our study. The age of all animals is indicated in the figure legends. Husbandry and ethical handling of mice were conducted according to guidelines approved by the Canadian Council on Animal Care. Genotyping was done by PCR using genomic DNA prepared from mouse ear punches.

### Histological and immunological analyses

Embryonic samples from timed matings (day of vaginal plug=E0.5) were collected, fixed in 4% paraformadehyde overnight (O/N) at 4 °C, serially dehydrated and then embedded in paraffin. Microtome sections of 7 μm thickness were examined histologically by periodic acid-Schiff staining. For immunofluorescent analysis, paraffin sections were dewaxed and serially re-hydrated with ethanol. Antigen retrieval was performed for 20 min in boiling Antigen Unmasking Solution (H-3300, Vector). Sections were incubated for 1 h in blocking solution (3% BSA, 10% goat serum, 0.1% Tween20 in PBS) at room temperature (RT). Blocking solution was replaced by a solution of primary antibodies diluted in 3% BSA, 3% goat serum and 0.1% Tween20 in PBS. The following primary antibodies were used in this study: CALBINDIN (PC253C, Calbiochem, 1:300), CITED1 (RB-9219-P0, Neomarkers, 1:200), CLEAVED-CASPASE 3 (#9661, Cell Signaling Technology, 1:300), CYTOKERATIN (C2562, Sigma, 1:200), CRUMBS3 (HPA013835, Sigma Prestige Antibodies, 1:300), E-CADHERIN (610181, BD Transduction Laboratories, 1:300), E-CADHERIN (#3195, Cell Signaling Technology, 1:300), ETV5 (13011-1-AP, Proteintech, 1:200), HNF4α (Gift from Marco Pontoglio, 1/300), LAMININ (L9393, Sigma, 1:300), LTL (FL-1321, Vector Laboratories, 1:300), NCAM (C9672, Sigma, 1:300), NF2 (HPA003097, Sigma Prestige Antibodies, 1:300), PAX2 (PRB-276P, Covance, 1:300), Phospho-AKT (#4060, Cell Signaling Technology, 1:50), Phospho-ERK (#4370, Cell Signaling Technology, 1:100), Phospho-YAP (#4911, Cell Signaling Technology, 1:150), SALL1 (AB31526, Abcam, 1:300), SIX2 (11562-1-AP, Proteintech, 1:300), SOX9 (AB5535, Chemicon, 1:300), YAP (sc-101199, Santa Cruz Biotechnology, 1:150), YAP (#14074, Cell Signaling Technology, 1:150), YAP/TAZ (#8418, Cell Signaling Technology, 1:150) and ZO-1 (#339100, Invitrogen, 1:500). Relevant conjugated secondary antibodies (Jackson Laboratories) were used for primary antibody detection. Slides were mounted using Vectashield with or without DAPI (Vector Labs). Fluorescent images were taken with a Nikon C1 plus Digital Eclipse confocal microscope.

### YAP overexpression in *Yap* ^*UB*^ mice

Pregnant mice were fed with a doxycycline diet (Teklad Custom Diet, TD.120769, 18% protein) containing 0.625 g kg^−1^ of doxycycline hyclate. Doxycycline hyclate contains ∼87% of doxycycline. This diet is designed to deliver a daily dose of 2–3 mg of doxycycline based on consumption of 4–5 g.

### Quantification of YAP^OE^ cells in mosaic kidneys

Quantification was done following immunostaining of E13.5 kidney paraffin section stained with antibodies against PAX2 and HA (11 867 423 001, Roche, 1:300). Ureteric tree was divided in three segments: UB tip cells (within 25 μm from the tip), the tip adjacent region (cells located 25–75 μm away from the tip) and the trunk (cells located 75 μm and more away from the tip). The number of HA-positive cells in each segment was quantified and divided by the total number of ureteric epithelial cells using an anti-PAX2 antibody. Numbers in brackets (Fig. 5i) represent the number of HA-positive cells per total number of UB PAX2-positive cells).

### Whole-mount immunofluorescence

Embryos were dissected at suitable time points and fixed in 4% PFA/PBS (pH 7.4) at 4 °C O/N then rinsed with PBS at RT several times. Urinary tracts were dissected under the microscope, soaked in PBS-BB (1% BSA, 0.2% skim milk, 0.3% Triton X-100 and 1 × PBS) to block and incubated O/N at 4 °C on a shaker. Tissues were incubated with primary antibody solution diluted in PBS-BB at 4 °C O/N, then washed with PBS-Tr (0.3% Triton X-100,1x PBS) twice at RT for 1–2 h and once at 4 °C O/N on a shaker. Tissues were incubated with secondary antibody diluted in PBS-BB O/N at 4 °C, washed several times in PBS-Tr at RT and imaged in 1 × PBS on a Nikon C1 confocal system with NIS Elements software (Nikon Instruments Inc., America).

### Kidneys explants

Kidney rudiments were dissected from E11.5 mouse embryos and place on filters (Millipore, 0.5 mm pore size) in direct contact with DMEM medium supplemented with 10% heat-inactivated foetal bovine serum, 10% glutamine and 1% penicillin/streptomycin. After 24 or 48 h (37 °C in 5% CO_2_), kidney rudiments were fixed in ice-cold methanol at 4 °C while still attached to their filters, washed in PBS and blocked for 1 h in 2% BSA/PBS at RT. Staining was performed using CALBINDIN antibody (PC253C, Calbiochem; 1:200 diluted in PBS, 0.1% BSA, 0.1% Triton) followed by detection with a Cy3-conjugated anti-rabbit antibody (1:150, Jackson Laboratories) and kidneys were examined using a fluorescence microscope.

To induce YAP overexpression in kidney explants, doxycycline (R&D Systems, AF2028) was added to the culture medium at different concentrations (1,500, 150, 30 and 15 ng ml^−1^) from day 1 in culture, unless stated otherwise.

### EdU incorporation

EdU solution containing 5-ethynyl-2′-deoxyuridine (10 mg ml^−1^) was injected intraperitoneally in pregnant mice (50 mg EdU kg^−1^ of mice) 10–15 min before embryonic dissection. The samples were prepared and sectioned as described above and analysed using the Click-iT EdU Alexa Fluor 488 Imaging Kit (Life Technologies).

### *In situ* hybridization

Anti-sense RNA probes labelled with digoxigenin (DIG, Roche) were prepared. Embryos were fixed in 4% paraformaldehyde in PBS O/N at 4 °C, paraffin embedded and cut at 7 μm and transferred onto superfrost glass slides. Fixed sections were hybridized O/N with antisense RNA probes at 65 °C in hybridization buffer. After several washes, sections were incubated in blocking buffer for 4 h, followed by incubation with anti-DIG antibody (Roche) in blocking buffer O/N at 4 °C. After several washes, the colorimetric reaction was carried out using BM Purple (Roche).

### Western blot

Western blot analysis was performed on stage E13.5 *Nf2*^*UB−/−*^ kidneys or cultured *Yap*^*UB-OE*^ kidneys following doxycycline induction. Unless stated otherwise each western blot lane represents one animal (two kidneys). Kidneys were mechanically homogenized and lysed in RIPA buffer supplemented with proteasome and phosphatase inhibitors. Western blot analysis was performed following standard protocols with the following primary antibodies: CALBINDIN (C9848, Sigma, 1:1,000), BAF155 (SC-9748, SantaCruz Biotechnology, 1:2,000), GAPDH (R9545, Sigma, 1:7,500), HA (11867423001, Roche, 1:1,000), LATS1 (#3477, Cell Signaling Technology, 1:1,000), MST1 (#3682, Cell Signaling Technology, 1:2,000), NF2 (HPA003097, Sigma Prestige Antibodies, 1:2,000), phosho-LATS1 S909 (#9157, Cell Signaling Technology, 1:500), phospho-MST1/2 T183/T180 (#3681, Cell Signaling Technology, 1:2,000), phospho-YAP S127 (#4911, Cell Signaling Technology, 1:2,000) and YAP (sc-101199, SantaCruz Biotechnology, 1:2,000). All uncropped western blots can be found in Supplementary Fig. 8.

### Cyst size quantification

Kidney masks were manually outlined in Fiji (PMID:22743772). Cyst segmentation was performed using Trainable Weka Segmentation v2.3.0 plugin for Fiji. The following features were used for pixel classification: Gaussian blur, difference of gaussians, Hessian, Sobel filter, variance and membrane projections. Objects were split in classes using Random Forest classifier. Classification was refined for each image to assure accurate segmentation. Measurements for individual cysts were further processed using MATLAB 2015b (Mathworks).

### Statistical analyses

All data are shown as mean values with s.d. An unpaired two-tailed *t*-test was used to determine differences between two groups (for example, wild type versus mutant). All statistical analyses were conducted using GraphPad Prism 5.0a software (La Jolla, USA).

### Data availability

The authors declare that all data supporting the findings of this study are available within the article and its Supplementary Information files or from the corresponding authors on reasonable request.

## Additional information

**How to cite this article:** Reginensi, A. *et al*. A critical role for NF2 and the Hippo pathway in branching morphogenesis. *Nat. Commun.* 7:12309 doi: 10.1038/ncomms12309 (2016).

## Supplementary Material

Supplementary Information Supplementary Figures 1-8

Supplementary Movie 1 Confocal z-stack imaging of Pax2 (red) and E-cadherin (green) staining in control E12.5 kidneys.

Supplementary Movie 2 Confocal z-stack imaging of Pax2 (red) and E-cadherin (green) staining in Nf2UB-/- E12.5 kidneys.
