# Supplementary material for: A critical role for NF2 and the Hippo pathway in branching morphogenesis
Source: Nat Commun. 2016 Aug 2;7:12309. doi: 10.1038/ncomms12309 (PMC4974664; doi:10.1038/ncomms12309)
Supplement: Supplementary Information — Supplementary Figures 1-8 [file ncomms12309-s1.pdf]

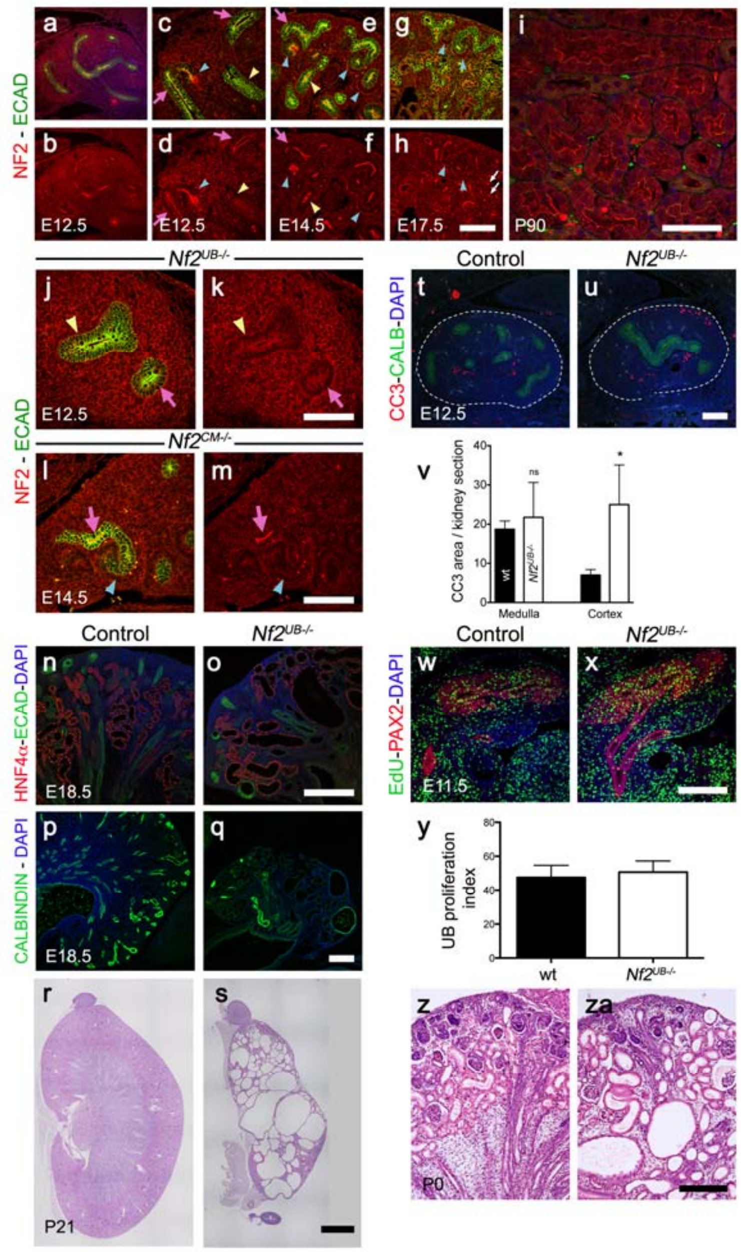

Supplementary Figure 1: *Nf2* deletion leads to kidney defects

(a-i) NF2 expression in the developing kidney at E12.5, E14.5, E17.5 and in adult nephrons (P90). Counterstaining with E-CADHERIN marks the UB and early nephrons. Pink arrows point to UB tips, yellow arrowheads to the ureter/CD, blue arrowheads to early nephrons and white arrows to proximal tubules. (j-m) Specificity of the NF2 apical staining was confirmed using *Hoxb7:Cre*$^{tg/+}$ (*Nf2*$^{UB-/-}$) and *Six2:Cre*$^{TGC/+}$ (*Nf2*$^{CM-/-}$) conditional deletion of *Nf2*. (n-q) Sections of E18.5 kidneys stained using late nephron markers confirms that dilatations are present in both nephron (proximal tubule: HNF4α) and collecting duct (CALBINDIN) compartments. (r,s) Parenchyma destruction in P21

Nf2$^{UB-/-}$ animals. (t,u) CLEAVED-CASPASE 3 staining in control and *Nf2*$^{UB-/-}$ kidneys at E12.5. Co-staining with CALBINDIN antibody highlights the UB compartment. (v) Quantification of CLEAVED-CASPASE 3 in medullary and cortical parts of controls and *Nf2*$^{UB-/-}$ mutants. Quantification of CLEAVED-CASPASE 3 was made on serial section from 6 kidneys per genotype. Error bars represent s.d., ns, not significant and $*P<0.05$, Student's *t*-test. (w-y) EdU incorporation in *Nf2*$^{UB-/-}$ and controls at E11.5 shows no change in proliferation (quantification in y). Co-staining with PAX2 antibody marks the UB cell nuclei. EdU quantification was made on 6 kidneys per genotype. A total of 628 and 807 cells were counted for control and *Nf2*$^{UB-/-}$. Error bars represent s.d. (z,za) Closer view of control and *Nf2*$^{UB-/-}$ kidneys at P0. Scale bars represent 100 µm (a-h, j-m, t,u), 50 µm (i), 200 µm (n-q, w-za) and 1 mm (r,s).

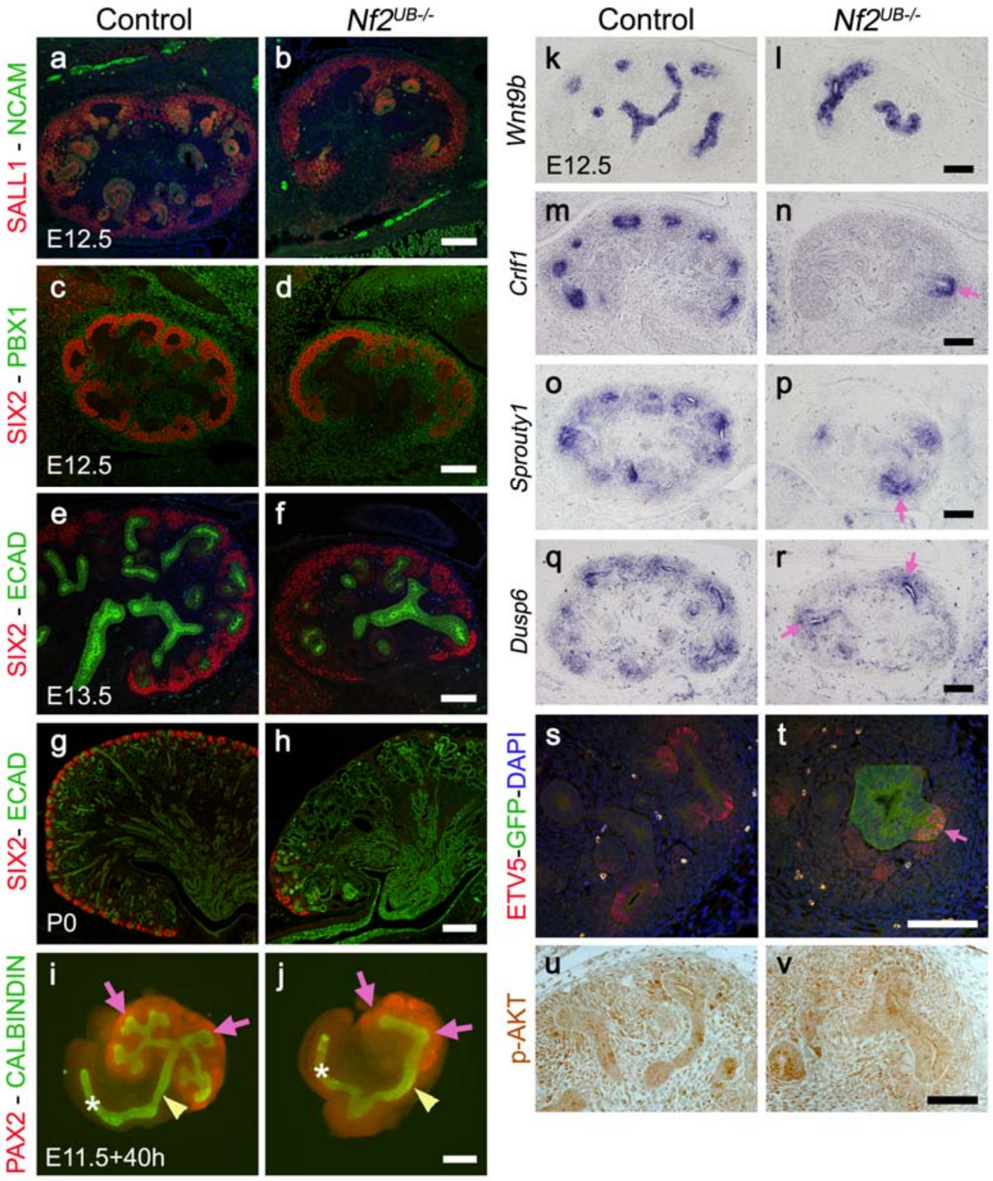

Supplementary Figure 2: *Nf2*$^{UB-/-}$ developmental characterization

(a-f) SALL1 and SIX2 staining showed reduce and sparse CM population in *Nf2*$^{UB-/-}$ mutants compared to controls at E12.5 and E13.5, while PBX1 reveals normal differentiation of the stromal compartment. (g,h) SIX2 staining indicates loss of the CM population in *Nf2* mutants at P0 compared to controls. (i,j) *Ex vivo* analysis confirms the reduced size of the CM population (marked by PAX2) upon *Nf2* loss. Pink arrows point to UB tips, yellow arrowheads to the ureter/CD and asterisks mark the Wolffian duct. (k,l) *Wnt9b* ISH shows conserved trunk specification in E12.5 *Nf2*$^{UB-/-}$ kidneys. (m-r) *Crlf1*, *Sprouty1* and *Dusp6* are expressed in UB tips (arrows) of *Nf2*$^{UB-/-}$ and control kidneys. (s,t) Co-staining for ETV5 and GFP confirms that expression of tip markers observed in *Nf2* mutants does not result from mosaic Cre expression. (u,v) Phospho-AKT staining at E12.5 is unchanged. Scale bars represent 100 µm (a-f, k-v), 200 µm (g,h) and 250 µm (i,j).

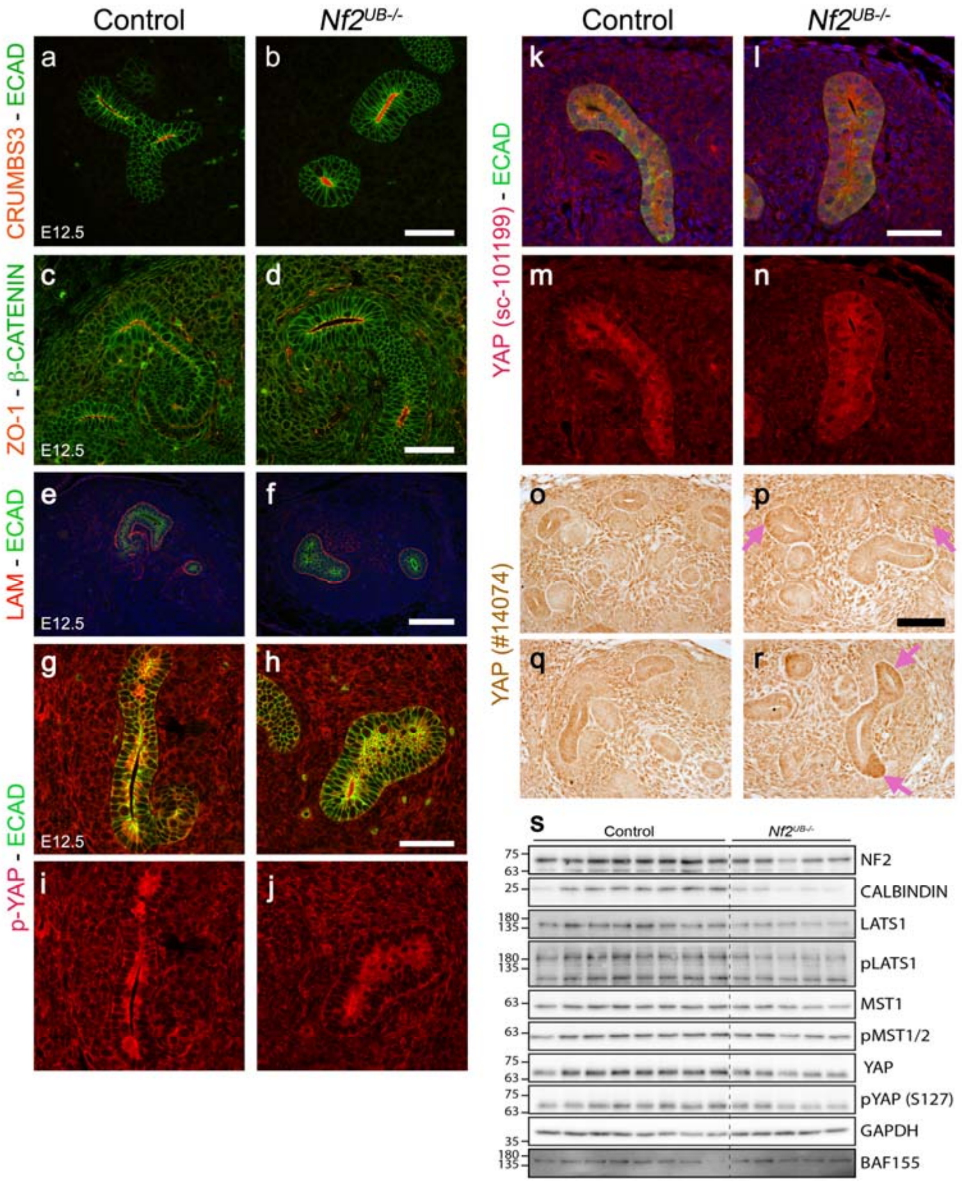

Supplementary Figure 3: Cell polarity and YAP/phospho-YAP staining in *Nf2$^{UB-/-}$*

Immunostaining for CRUMBS3/E-CADHERIN (a,b), ZO-1/β-CATENIN (c,d) and LAMININ (e,f) reveals no major cell polarity defects in the UB compartment of *Nf2$^{UB-/-}$* mutants compared to controls at E12.5. (g-r) No changes in YAP and phospho-YAP levels or subcellular localization are observed in the UB compartment of *Nf2$^{UB-/-}$* mutants compared to controls at E12.5. (s) Western blot analysis of lysates from E13.5 control and *Nf2$^{UB-/-}$* kidneys (two kidneys par lane). Scale bars represent 50 µm (a-d, g-n) and 100 µm (e,f, o-r).

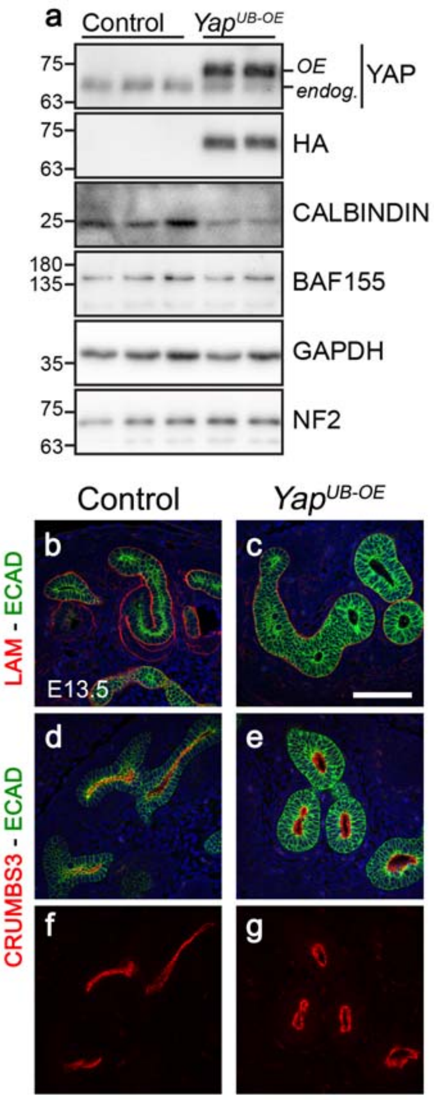

Supplementary Figure 4: YAP overexpression leads to kidney hypoplasia
(a) Western blot analysis of E11.5 controls and *Yap*$^{UB\text{-}OE}$ explants (6 kidneys pooled for each lane) cultured for 24 hours in presence of 1500 ng/ml of doxycycline. These results validate the experiment system, as *Yap*$^{UB\text{-}OE}$ explants show increased YAP expression (also HA positive) and reduced CALBINDIN expression as a result of loss of branching. No change was observed in NF2 levels excluding a feedback loop. BAF155 and GAPDH serve as loading controls. (b-g) Immunostaining for LAMININ, CRUMBS3 and E-CADHERIN reveals no major defects in cell polarity in the UB compartment of *Yap*$^{UB\text{-}OE}$ mutants compared to controls at E13.5. Scale bar represent 100 µm (b-g).

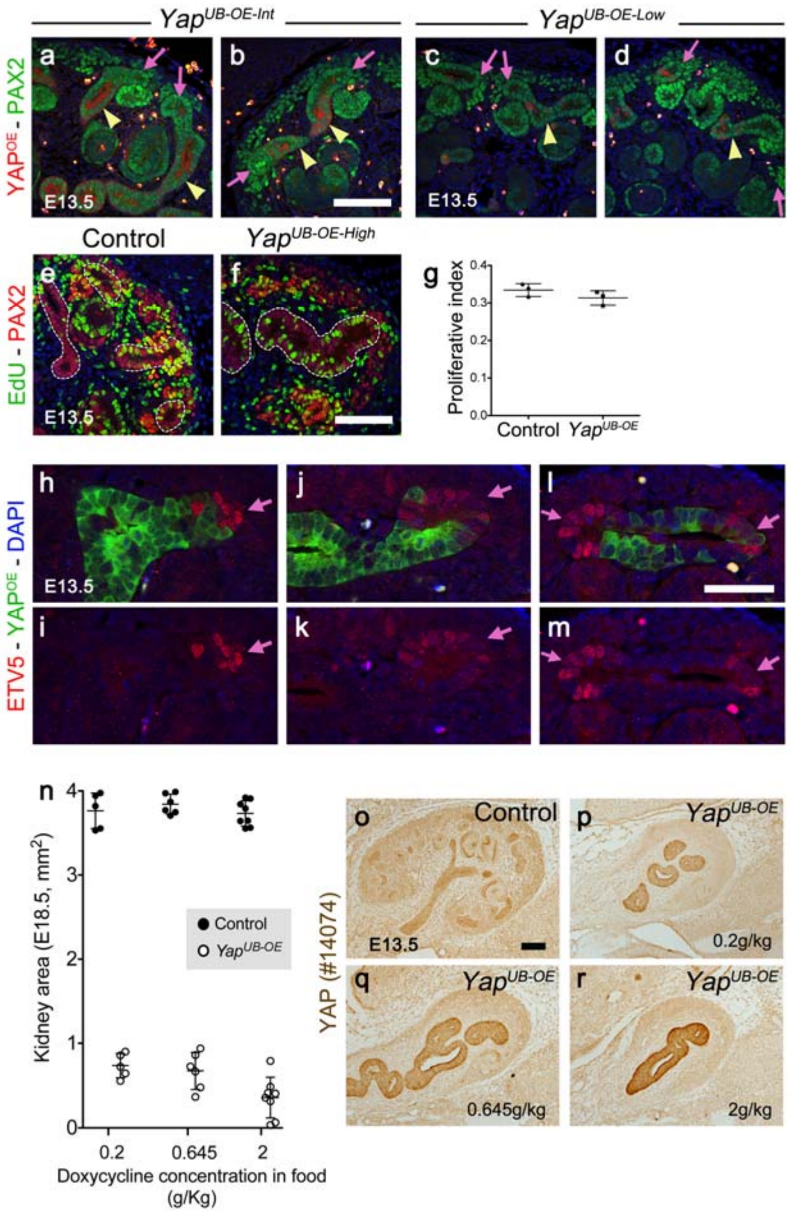

Supplementary Figure 5: Mosaic analysis of YAP overexpression

(a-d) Examples of the distribution of YAP^{OE} cells in low and intermediate *Yap* mutant animals. Pink arrows point to UB tips and yellow arrowheads to trunks. (e-g) Analysis of proliferation (EdU incorporation) in *Yap*$^{UB\text{-}OE}$ mutants and controls. EdU quantification was made on 3 embryos for control (455 cells counted) and *Nf2*$^{UB-/-}$ (518 cells counted). Error bars represent s.d. (h-m). Single channel images of the panels show in Figure 5j-l. (n) Quantification of kidney size of control and *Yap*$^{UB\text{-}OE}$ embryos at E18.5 after feeding pregnant dams with different doxycycline diets from E11 to E18.5. (o-r) YAP staining at E13.5 in embryos exposed to different concentrations of doxycycline. Scale bars represent 100 µm (a-f, o-r) and 50 µm (h-m).

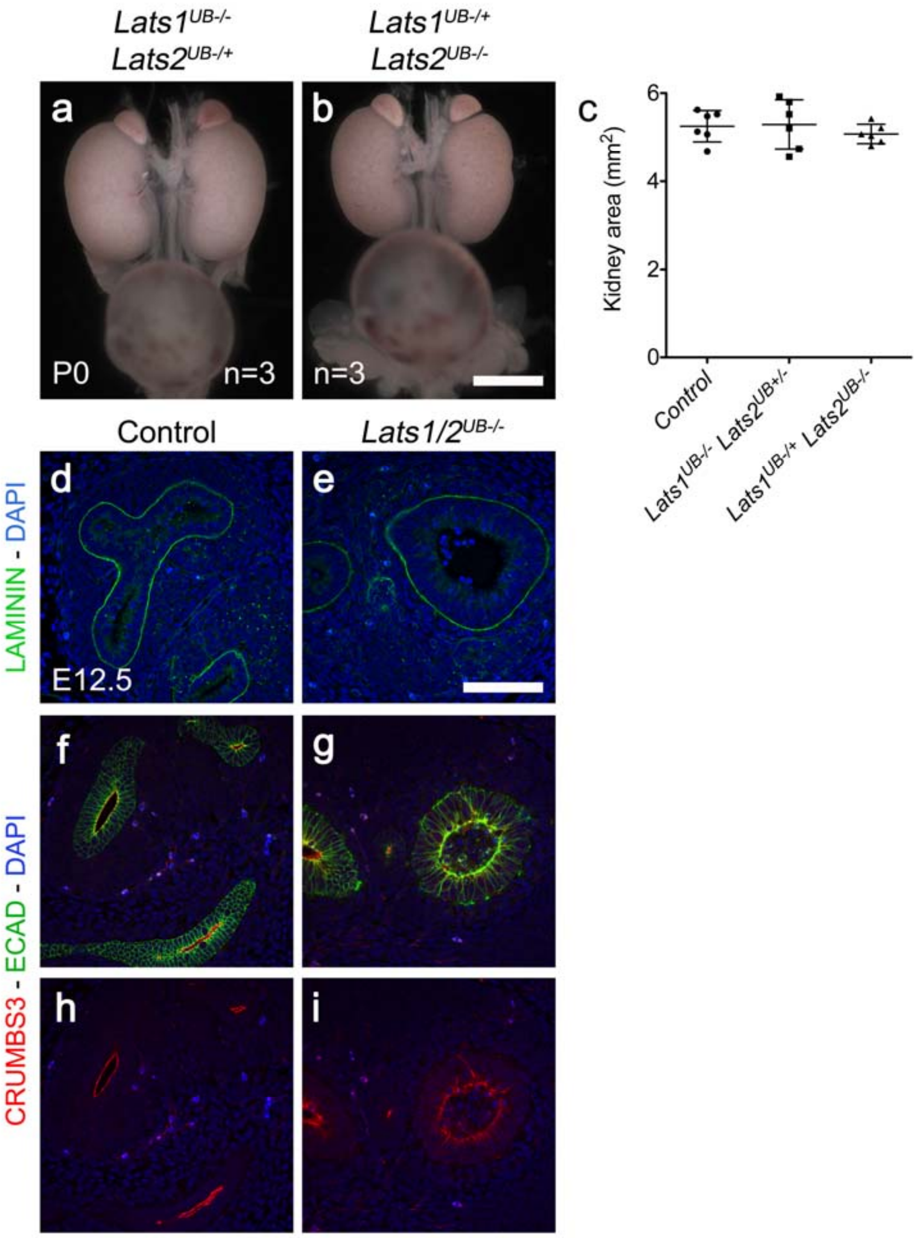

Supplementary Figure 6: *Lats1/2* deletion in the UB compartment leads to cell polarity defects

(a,b) Macroscopic views of the urogenital system of $Lats1^{UB-/-}$ $Lats2^{UB-/+}$ and $Lats1^{UB-/+}$ $Lats2^{UB-/-}$ newborns. (c) Quantification of kidney size (6 kidneys for each genotype) reveals that deletion of three out of four *Lats1* and *Lats2* alleles does not substantially affect kidney size regardless of allelic combinations. (d-i) Polarity defects are observed in the UB compartment of $Lats1/2^{UB-/-}$ kidneys compared to controls using LAMININ (d,e), CRUMBS3 and E-CADHERIN (f-i) staining at E12.5. Scale bars represent 1 mm (a,b) and 100 µm (d-i).

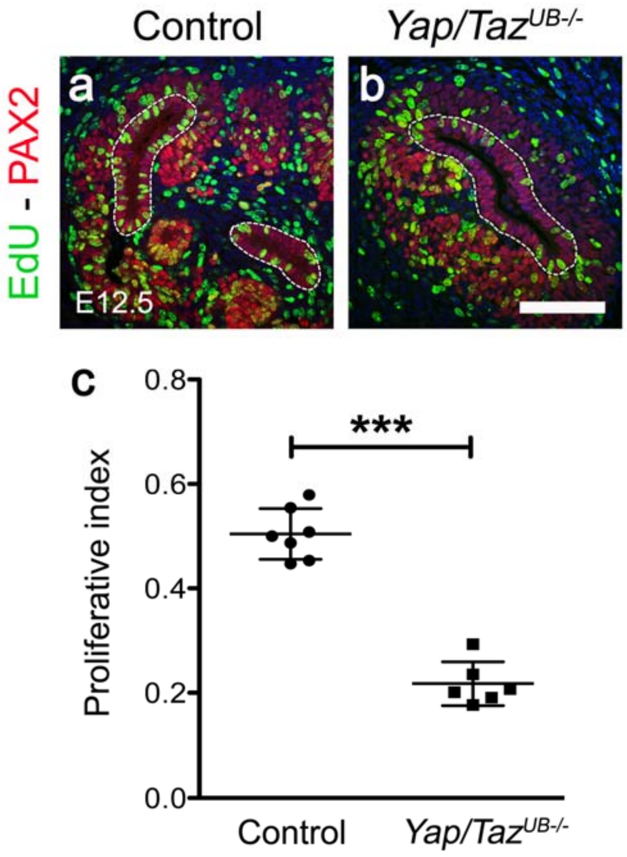

Supplementary Figure 7: Reduced proliferation in UBs depleted of *Yap* and *Taz*
(a,b) EdU incorporation in the UB compartment (counterstained with PAX2 and outlined with a dashed line) of *Yap/Taz*$^{UB-/-}$ mutants and controls at E12.5. (c) Quantification of proliferative index of UB cells (percentage of EdU positive cells / total of PAX2 cells) reveals a significant decrease in proliferation in *Yap/Taz* mutants compared to controls. EdU quantification was made on 3 embryos for control (547 cells counted) and *Yap/Taz*$^{UB-/-}$ (862 cells counted). Error bars represent s.d., ***P<0.0001, Student's *t*-test. Scale bar represents 100 µm.

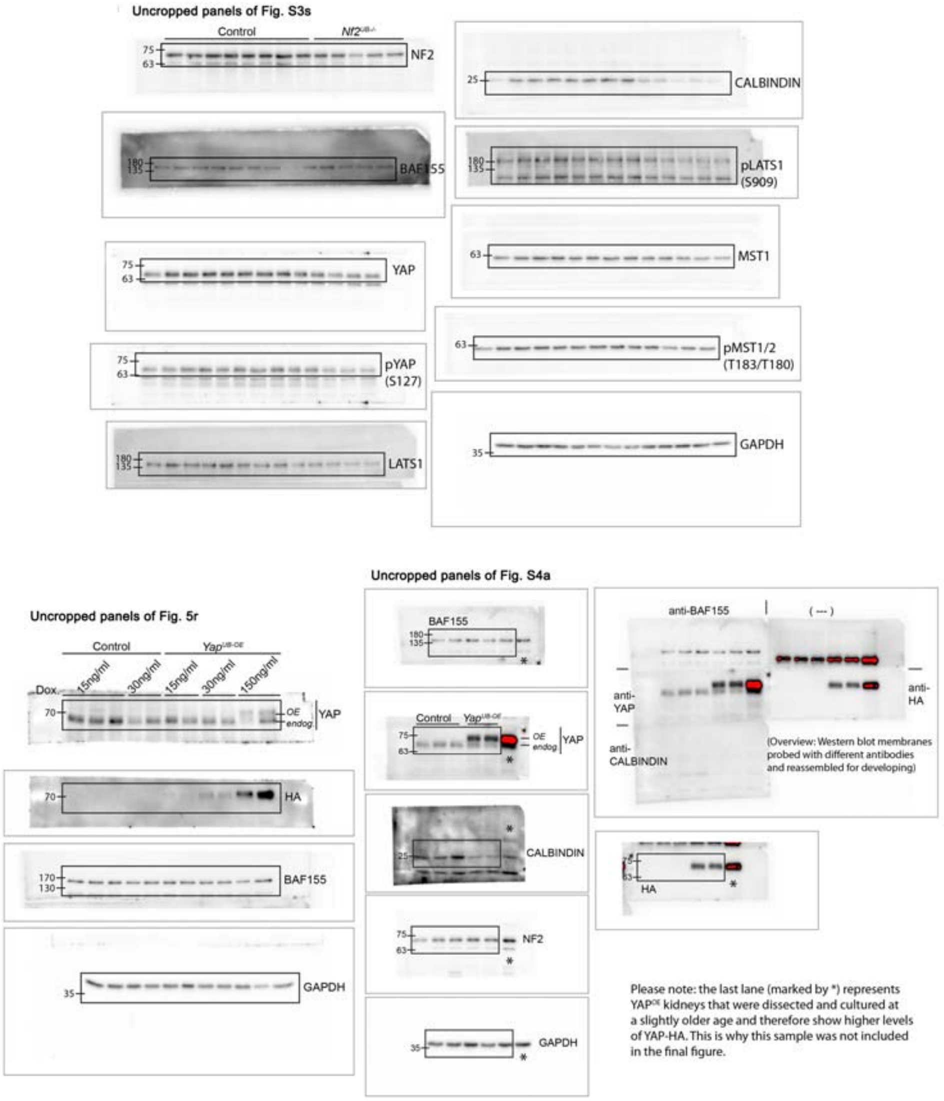

Supplementary Figure 8: Uncropped Western blots
